# Averaged kick maps: less noise, more signal…and probably less bias

**DOI:** 10.1107/S0907444909021933

**Published:** 2009-08-06

**Authors:** Jure Pražnikar, Pavel V. Afonine, Gregor Gunčar, Paul D. Adams, Dušan Turk

**Affiliations:** a Jožef Stefan Institute, Slovenia; b Lawrence Berkeley National Laboratory, Berkeley, USA; c Department of Bioengineering, University of California, Berkeley, USA

**Keywords:** kick maps, OMIT maps, density-map calculation, model bias, maximum likelihood

## Abstract

Use of reliable density maps is crucial for rapid and successful crystal structure determination. Here, the averaged kick (AK) map approach is investigated, its application is generalized and it is compared with other map-calculation methods. AK maps are the sum of a series of kick maps, where each kick map is calculated from atomic coordinates modified by random shifts. As such, they are a numerical analogue of maximum-likelihood maps. AK maps can be unweighted or maximum-likelihood (σ_A_) weighted. Analysis shows that they are comparable and correspond better to the final model than σ_A_ and simulated-annealing maps. The AK maps were challenged by a difficult structure-validation case, in which they were able to clarify the problematic region in the density without the need for model rebuilding. The conclusion is that AK maps can be useful throughout the entire progress of crystal structure determination, offering the possibility of improved map interpretation.

## Abstract 2

Averaged kick maps are the sum of a series of individual kick maps, where each map is calculated from atomic coordinates modified by random shifts. These maps offer the possibility of an improved and less model-biased map interpretation.

## 1. Introduction

After crystallographic phases have been obtained, an iterative procedure is used to cycle through density-map calculation, molecular model building, rebuilding and refinement until the consistency of the model with the experimentally measured structure factors is maximized. When experimental phases are available, they provide a source of phasing information that is independent of the model. However, in the molecular-replacement case (Rossmann, 1972 ▶) the model is the sole source of phasing information which, by transformation into density maps, guides model building and rebuilding (Watenpaugh *et al.*, 1973 ▶). Here, we focus on density-map calculation where a prior molecular model is used as the sole source of phasing, although the proposed procedure can also be applied to *de novo* structure determination. The density maps have the potential to reveal more information than is provided by the current working model. Simultaneously, they are the source of misleading information: it is typically the case that the molecular-replacement models used for phasing may be partially incorrect and thus bias the resulting maps. Sometimes a thin line separates the correct interpretation of a density map from an incorrect interpretation. Therefore, it is important to derive maps which assure that the model modifications suggested by map interpretation indeed converge towards the true structure.

Throughout the history of crystal structure determination, a significant amount of effort has been directed into the development of density-map calculations with the aim of enhancing the signal and reducing errors and noise (Luzzati, 1953 ▶; Woolfson, 1956 ▶; Sim, 1959 ▶; Raman, 1959 ▶; Ramachandran & Raman, 1959 ▶; Srinivasan, 1961 ▶; Ramachandran & Srinivasan, 1961 ▶, 1970 ▶; Main, 1979 ▶; Vijayan, 1980 ▶). Read (1986 ▶) generalized these approaches and proposed that the least biased maps be computed as (2*m*|*F*_obs_| − *D*|*F*_model_|)exp(*i*ϕ_model_) for non­centric data and |*F*_obs_|exp(*i*ϕ_model_) for centric data; these maps should correspond maximally with the true map. A further improvement towards reducing map bias was the computation of weighting terms (*m* and *D*) using a subset of reflections set aside for cross-validation (‘test’ reflections; Brünger, 1992 ▶) and used for estimation of phase errors (Lunin & Skovoroda, 1995 ▶; Pannu & Read, 1996 ▶; Urzhumtsev *et al.*, 1996 ▶). More sophisticated approaches, such as the use of multi-start simulated annealing (Hodel *et al.*, 1992 ▶; Rice *et al.*, 1998 ▶), also provide improved estimates for the error model and map weights. As low-resolution data are important for map quality, a series of bulk-solvent correction models have been developed in order to be able to include all reflections in the map calculation rather than applying a low-resolution truncation (Moews & Kretsinger, 1975 ▶; Phillips, 1980 ▶; Jiang & Brünger, 1994 ▶; Tronrud, 1997 ▶; Badger, 1997 ▶; Urzhumtsev, 2000 ▶; Fokine & Urzhumtsev, 2002 ▶). Crystal anisotropy can severely distort the map appearance so an anisotropic correction is typically used (Sheriff & Hendrickson, 1987 ▶; Murshudov *et al.*, 1998 ▶; Usón *et al.*, 1999 ▶). All these advances have been combined in efficient and robust model structure-factor (*F*_model_) calculation protocols such as those of Afonine *et al.* (2005*b* ▶) and Brunger (2007 ▶). Averaging of either electron-density maps or structure factors has a long tradition in crystallography, starting with noncrystallographic symmetry averaging as first described by Rossmann & Blow (1963 ▶) and progressing to averaging of maps and structure factors generated from several (Perrakis *et al.*, 1997 ▶) or multiple models (Hodel *et al.*, 1992 ▶; Terwilliger *et al.*, 2007 ▶). The averaged kick (AK) map method that we describe here is a complementary approach to enhancing the signal and reducing the noise in maps calculated from molecular models with errors.

More than a decade ago, during the final stages of the determination of the crystal structure of porcine cathepsin H (Gunčar *et al.*, 1998 ▶), the question of the directionality of the binding of the eight-residue-long propeptide attached to the body of the enzyme by a disulfide bond, termed the mini-chain, prompted the development of new methods to maximize map quality. Visual interpretation of the map suggested that the mini-chain has to be positioned in the reverse direction compared with the propeptide as expected from previous proenzyme structures such as cathepsin B (Turk *et al.*, 1996 ▶). Neither the inspection of the unweighted difference map com­bined with refinement of the mini-chain traced in two alternative directions nor the use of simulated annealing in refinement were able to deliver maps which unambiguously revealed the directionality of the mini-chain.

We hypothesized that if atoms were displaced by independent random shifts then the correlations of atomic positions imposed through refinement *via* structure factors and chemistry terms would be lost. As a result, random shifts of co­ordinates, termed kicking, was introduced into the program *MAIN* (Turk, 1997 ▶, 2007 ▶; Gunčar *et al.*, 1998 ▶) and applied to map calculations and refinement. The question was asked: ‘What would happen when a series of kick maps were averaged?’ (T. Terwilliger, personal communication). The resulting procedure appears to be an analogue of the maximum-likelihood (ML) approach, as pointed out by an anonymous referee: the crystallographic ML theory supposes that the current model can be corrected by introducing random errors and suggests structure-factor correction after ‘theoretical averaging’ of such models with random shifts. It has been demonstrated that these maps are an improvement over single kick maps (Gunčar *et al.*, 2000 ▶; Than *et al.*, 2002 ▶, 2005 ▶). Additionally, the concept of a second-generation averaged kick map was introduced (discussed later). The latter approach was found to reveal the problem areas of a structure, allowing rebuilding and subsequent refinement (Fernandez-Catalan *et al.*, 1998 ▶). In this work, we have analyzed the kick-map method and its potential and have optimized and generalized the procedure for its use. Com­parison with other maps such as unweighted (UN), maximum-likelihood weighted (ML) and simulated annealing (SA) maps show that the averaged kick (AK) map has the potential to reduce or eliminate model bias and can be a useful alternative scheme for map calculations at any stage of structure solution.

## 2. Methods and models

### 2.1. Software applications

All map calculations and their comparisons were performed with the crystallographic program *MAIN* (Turk, 1992 ▶), with the exception of the simulated-annealing runs, which were performed using the *phenix.refine* program (Afonine *et al.*, 2005*a* ▶) of the *PHENIX* package (Adams *et al.*, 2002 ▶).

### 2.2. Electron-density maps

We used the following maps for all tests discussed below.

(i) |*F*_model_|exp(*i*ϕ_model_) maps (referred to as *F*_model_ maps in the following).
(ii) Simple unweighted maps (2|*F*_obs_| − |*F*_model_|)exp(*i*ϕ_model_), (|*F*_obs_| − |*F*_model_|)exp(*i*ϕ_model_) (2*F*_obs_ − *F*_model_ or *F*_obs_ − *F*_model_).
(iii) Maximum-likelihood weighted (2*m*|*F*_obs_| − *D*|*F*_model_|)exp(*i*ϕ_model_), (*m*|*F*_obs_| − *D*|*F*_model_|)exp(*i*ϕ_model_) (2*F*_obs_ − *F*_model_ or *F*_obs_ − *F*_model_).
(iv) Simulated-annealing maps (SA).
(v) UN and ML averaged kick maps (UN AK, ML AK).

*F*_model_ is the total model structure factor that accounts for all atoms and the bulk-solvent contribution, as well as various scales (as defined, for example, in Afonine *et al.*, 2005*b* ▶). The final *F*_model_ maps generated from the best available structures were used as the reference. The coefficients of the ML maps (Read, 1986 ▶; Pannu & Read, 1996 ▶; Murshudov *et al.*, 1997 ▶) were computed as described in Lunin & Skovoroda (1995 ▶) and Urzhumtsev *et al.* (1996 ▶). The SA maps were calculated from an ensemble of models generated by running a number of SA refinements, each starting with the same input model and a different random seed (Rice *et al.*, 1998 ▶). The final SA map is the average of all maps computed from individual models. The AK map calculation is described below.

The kicked maps are based on structural models with randomly displaced atoms. Each kick modifies the atomic coordinates by a random shift within a given interval for which each point within a cube around the atomic position has an equal probability for the new position. Structure factors are calculated from the ‘kicked’ atomic model and used for map calculation, mostly of 2*F*_obs_ − *F*_model_-type maps. After each map calculation the model coordinates are restored to their original values. The final AK map is the average of the whole series of kick maps, where *F*_model_ for each map is calculated from the kicked atomic positions generated with a different random number seed. Each time the model is kicked, the starting seed is changed to produce a novel series of random numbers that is unique for each consecutive model modification. In this scheme, the |*F*_model_| are scaled to |*F*_obs_| using the unweighted or maximum-likelihood weighting schemes. For ML AK maps, the *m* and *D* values computed from the original (undisturbed) model are used in all subsequent individual kick-map calculations. (The coefficients derived from the distorted models result in values which can produce substantially worse maps.) Analogously, the bulk solvent *k*_sol_ and *B*_sol_ parameters and bulk-solvent structure factors as well as the anisotropic scale matrix should be retained from the original model and used when computing *F*_model_ for kicked structures.

There are two methods for AK map calculation: one can average the *F*_model_ structure factors and calculate the map using the averaged *F*_model_ or average the individual kick maps,
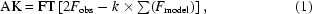

               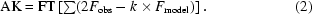
The principal difference between the two is the following: in the first case scaling of |*F*_model_| to |*F*_obs_| is performed once for the set of averaged *F*_model_ and the 2*F*_obs_ − *F*_model_ map is computed (1), whereas in the second case each individual |*F*_model_| from the series is scaled to |*F*_obs_| and an individual 2*F*_obs_ − *F*_model_ map is computed, added to the sum of the maps and averaged at the end. The latter approach has been found to be superior and is used throughout this work (data not shown). To speed up the process and since the Fourier transformation (FT) is a linear operation, the averaging of maps in real space can be replaced by the averaging of corresponding Fourier coefficients followed by a single map calculation.

### 2.3. Map comparison

To compare the maps, we used overall and local map correlation co­efficients (CCs) as commonly used (Lunin & Woolfson, 1993 ▶; Lunin & Skovoroda, 1995 ▶) and the density at positions of the atomic model. For the overall CC the whole unit cell was used, whereas for the CC of the local map the region around part of the atomic model was selected. A CC of greater than 0.8 is generally described as a strong correlation, whereas a CC of less than 0.5 is a weak correlation (Lunin & Woolfson, 1993 ▶; Lunin & Skovoroda, 1995 ▶).

The density at the positions of the atoms was obtained by linear interpolation of the density values from the eight surrounding grid points.

### 2.4. The second-generation maps

After the electron-density map of 2*F*_obs_ − *F*_model_ type has been calculated from the working model (the first-generation map), the parts of the model that are inconsistent with the map are omitted from the next map calculation. Practically, the occupancy of atoms is assigned in accordance with the first-generation map. The procedure in *MAIN*, termed ‘AUTO_WEIGHT’, checks all atoms of every residue in the model starting from the root atom onwards to the end of the side chain. As long as the atoms lie in density above the threshold value (the default is 1.0σ) their occupancy is set to 1.0, otherwise the search is terminated and the nonchecked atoms receive zero occupancy. The resulting map is termed the second-generation map. The second-generation map can be calculated using any kind of 2*F*_obs_ − *F*_model_ map.

### 2.5. Crystal structures

As study cases, we have used the molecular-replacement solutions of stefin B (PDB code 2oct; Jenko *et al.*, 2007 ▶), cathepsin H (PDB code 8pch; Gunčar *et al.*, 1998 ▶), ammodytin L (PDB code 3dih; D. Turk, G. Gunčar & I. Krizaj, unpublished work) and three additional cases from the PDB, namely PDB codes 2ahn (Y. Dall’Antonia, T. Pavkov, H. Fuchs, H. Breiteneder & W. Keller, unpublished work), 2fy2 (Kim *et al.*, 2006 ▶) and 1twl (Southeast Collaboratory for Structural Genomics, unpublished work). The statistics for the diffraction data and models are provided in Table 1 ▶. Molecular-replacement solutions were either taken from the original works or found using *EPMR* (Kissinger *et al.*, 1999 ▶).

## 3. Results

To validate AK maps, we considered addressing the following questions.

(i) Which AK key calculation parameters (kick size, number of averaged maps) are optimal under different circumstances (resolution range, correctness of the model)?
(ii) Are the AK maps a reasonable alternative to UN, ML and SA maps?
(iii) Can the AK maps provide an improved interpretation leading to a better model?

Since we were comparing different maps and not seeking errors in models, we have chosen the final *F*_model_ phases and amplitudes with the addition of bulk-solvent correction as the target since they represent the true solution of the structure.

### 3.1. How to calculate the best AK map

#### 3.1.1. How many kick maps should be averaged to achieve convergence?

**Figure 1:**
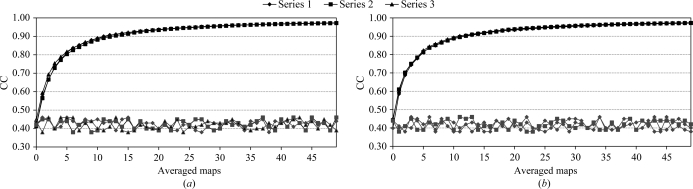
Convergence of AK maps depending on random number seeds. The upper smooth curves show the CCs of pairs of AK maps as a function of the number of maps averaged, whereas the bottom curves plot the CC of each individual map compared with the final map of the series. Three series of AK maps were calculated from three different starting random number seeds to avoid any map repetition. The applied kick size was 0.9 Å at a resolution of 3.0 Å. The crystal structures of P79S stefin B variant and cathepsin H were used as initial models in (*a*) and (*b*), respectively.

To find out how many kick maps are required for convergence (where the average map does not change upon the addition of one more kick), we compared three series of AK maps using two different cases (Table 1 ▶). Each series of maps was generated with a starting random seed separated sufficiently to exclude the repetition of any kick map (Fig. 1 ▶). The increases in the CC of the partial sums of the three series overlap so closely that they are indistinguishable, whereas the CC of each map in the series, when compared with the final AK map, fluctuates around the starting pairwise correlations between the three series of around 0.4–0.5. The CCs of the partial sums increase rapidly towards 0.8. After approximately ten averaged maps the CCs are already at 0.9 and converge after 40 averaged maps to a CC of approximately 0.97–0.98. Thus, at least 40 individual kick maps, the similarity of the CCs of which to the final map of the series fluctuates within the ±0.05 interval, are required to result in a convergent and reproducible AK map. An equivalent com­parison has also shown that the series of AK maps converges towards the correct map. (The plots are not shown since they appear equivalent to those in Fig. 1 ▶, only with a lower final CC.)

#### 3.1.2. Which kick size to use

Clearly, a small kick size produces structure factors that are similar to those derived from the unperturbed model and a very large kick size produces meaningless structure factors. The optimal kick size may be dependent on the resolution range of the diffraction data used and the quality of the structural model itself. We inspected this dependence using models saved in the course of determination of the crystal structure of cathepsin H.

**Figure 2:**
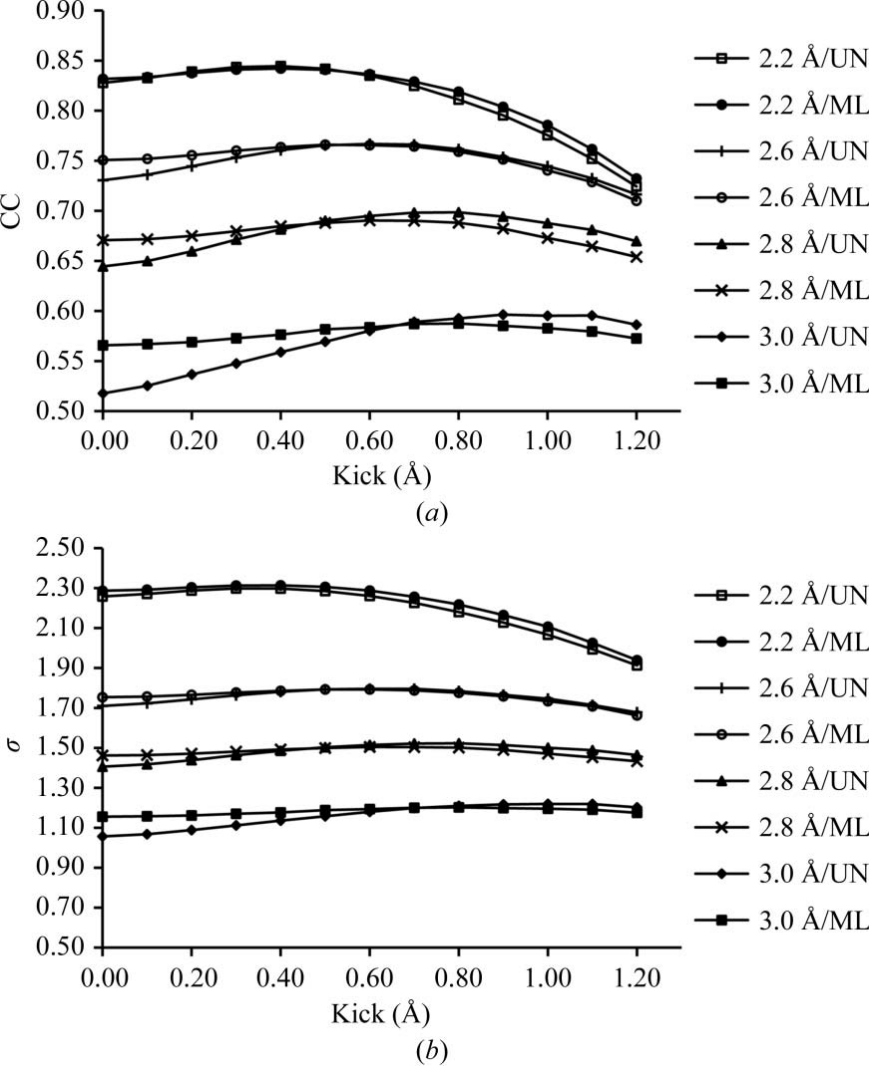
Map improvement as a function of kick size and model quality. The UN AK and ML AK maps calculated from four molecular models corresponding to four different stages (see Table 2 ▶) in the determination of the crystal structure of cathepsin H. For each model UN AK and ML AK maps were calculated with kick sizes from 0.1 to 1.2 Å from 100 kick maps. Zero kick size corresponds to the UN and ML maps, respectively. In (*a*) CCs between the final *F*_model_ map and UN AK and AK maps are shown. (*b*) shows the average density of the final model atoms in each particular map.

AK maps were calculated from four models saved at various steps of structure determination corresponding to four different high-resolution limits in the range 3.0–2.2 Å. Their *R* factors range from 0.43 to 0.25 and the r.m.s. deviations of their C^α^ atoms from the final model range from 0.91 to 0.27 Å (Table 2 ▶), reflecting the increasing similarity of the models to the refined structure. The smallest kick size applied was 0.1 Å and the largest was 1.2 Å (Fig. 2 ▶). Fig. 2 ▶(*a*) shows that with an increase in the kick size the AK maps start approaching the final *F*_model_ map. After reaching a rather broad peak of optimal kick size their similarity to the final *F*_model_ map begins to decrease. The map improvement is larger (0.03) for earlier models than with model 4 (0.01). This behaviour is shared by both UN AK and ML AK maps. The ML AK maps start higher and show a smaller increase than the UN AK maps. An equivalent picture is revealed by the density at positions of the final model (Fig. 2 ▶ *b*). The highest increase in average density at positions of the final model in the AK maps is increased by 0.06, 0.06, 0.04 and 0.01 σ levels, respectively. As expected, concomitant with the progress of structure determination and the increase of the upper limit of diffraction data resolution, the optimal kick size decreases. For the lowest resolution model 1 (3.0 Å resolution) it is about 1.0 and for models 2, 3 and 4 it is 0.8, 0.6 and 0.4 Å, respectively. The optima are rather broad. The optimal kick size depends on the model quality and only marginally depends on the resolution range of the data applied (data not shown), which is consistent with the expectations from the ML error estimates (Table 3 ▶).

This brief analysis suggests that the optimal kick size should be adjusted for each particular case. Interestingly, the optimal kick sizes are not far from the ML-based coordinate error estimates, thus confirming the analogy between the AK map and ML approaches. To avoid the need for a decision on the kick size, a series of AK maps using kick sizes in the range from 0.1 to 1.0 was calculated and averaged. The resulting maps deliver CCs (0.58, 0.69, 0.77, 0.84) which are close to the best AK map from the series (0.60, 0.70, 0.77, 0.84) and are approximately the same for the UN AK and ML AK maps. This generalizes the AK map-calculation setup and makes it more broadly applicable.

### 3.2. Comparison with other types of maps

The structure determination of molecules using molecular replacement can be described by three basic phases: initial, intermediate and final.

The quality of the AK maps was explored and compared with the final *F*_model_ map in parallel with the other available map types: UN, ML and SA. Comparisons were performed on the global level, taking into account all density points in the unit cell, as well as locally around selected regions of the molecular models.

#### 3.2.1. Maps from starting models

In the initial phase, the search model positioned by molecular replacement is used to calculate the electron-density map. To demonstrate the use of AK maps, we have calculated molecular-replacement solutions and maps for cathepsin H (PDB code 8pch; Gunčar *et al.*, 1998 ▶), ammodytin L (PDB code 3dih; D. Turk, G. Gunčar & I. Krizaj, unpublished work), stefin B (PDB code 2oct; Jenko *et al.*, 2007 ▶) and three additional cases from the PDB, namely 2ahn (Y. Dall’Antonia, T. Pavkov, H. Fuchs, H. Breiteneder & W. Keller, unpublished work, Table 1 ▶), 2fy2 (Kim *et al.*, 2006 ▶) and 1twl (Southeast Collaboratory for Structural Genomics, unpublished work), using actinidin (Baker, 1980 ▶), *Crotalus atrox* phospholipase A_2_ (Keith *et al.*, 1981 ▶), 1thv (Ko *et al.*, 1994 ▶), 1q6x (Cai *et al.*, 2004 ▶) and 1ude (Liu *et al.*, 2004 ▶) as search models. The maps were calculated from the search models using all protein atoms.

**Figure 3:**
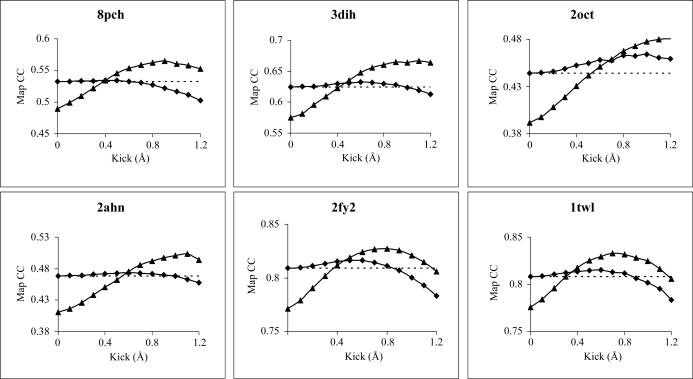
AK maps derived from 100 kick maps were calculated at 3.0 Å resolution for molecular-replacement solutions of cathepsin H (PDB code 8pch), ammodytin L (3dih), stefin B tetramer (2oct) and three other structures from the PDB (2ahn, 2fy2 and 1twl), using actinidin, *C. atrox* phospholipase A_2_, 1thv, 1q6x and 1nde as search models. The graphs represent the CCs between the *F*_model_ map of the final refined structures and the AK map (UN AK, triangles; ML AK, circles) of the molecular-replacement solutions at different kick step. The dashed straight line represents the CC between the *F*_model_ of the final structure and the 2*F*_obs_ − *F*_calc_ ML map of the molecular-replacement solution without kicks.

Maps were compared with the final *F*_model_ map. The obtained CCs are plotted in Fig. 3 ▶ using the CC coefficient of the ML map to denote the aimed value. The graphs reveal that UN AK and ML AK maps can deliver maps that are closer to the final *F*_model_ map than the ML map. The map improvements in the series correspond to an increase in the CC of up to 0.04. The average map of series of AK maps from 0.1 to 1.0 Å kick size delivers a somewhat lower increase in CC (0.02). In cases where omission of atoms inconsistent with the map was particularly successful, an increase in the CC of the second-generation maps was also observed (0.02 for 2ahn, 0.02 for 1twl, 0.02 for ammodytin L) despite the reduced scattering power of the remaining parts of the molecular model.

Another insight into the properties of the AK maps is provided by the match of the final model to the initial maps equivalent to Fig. 2 ▶(*b*) (data not shown). Density values were calculated at the positions of all protein atoms of the final model for each map and averaged. When compared with the density delivered by the ML map, the averaged series of AK maps for all six cases show improved positioning of the final model in the map, with the highest average increase of 0.09σ in the case of ammodytin L. Clearly, this average gain in the maps indicates that local map improvements were even more significant.

These cases indicate that AK maps can produce maps that are closer to the final solution and may have the potential to reveal map features that are otherwise inaccessible by map calculation alone, as manifested in the case below. However, the map improvements are not uniformly distributed: they are position-dependent and case-dependent.

#### 3.2.2. Maps from the intermediate phase

In the intermediate phase, the models are partially refined and more or less complete. They still contain regions with errors and lack flexible loops and ligands. The positions of the residues still need to be examined and adjusted to best fit the electron-density maps.

To recreate a typical situation from the middle of structure determination, multiple SA refinement runs were performed, resulting in 30 models with crystallographic *R* values of around 0.30. None of the models contained solvent or ligands and none were entirely correct. However, the models contained no sequence frame shifts. For the map comparison, we picked one of the models and used it as an input for UN, ML and AK map calculation, whereas all 30 models were used as input for SA map calculation. The following map comparisons with the final *F*_model_ maps are based on the whole unit cell as well as along the chain of residues. While the whole unit-cell comparisons provide a measure of the overall quality of the map, the local comparisons provide insight into individual features.

*Maps from the intermediate phase: global comparison for the whole unit cell.* Table 3 ▶ shows that the AK, ML and SA maps are rather similar to the final *F*_model_ map, with CCs in the ranges 0.84–0.85 and 0.80–0.81 for the ammodytin L and cathepsin H cases, respectively. The CCs of the whole unit-cell UN maps compared with the *F*_model_ maps are about 0.02–0.03 lower (Table 3 ▶). The differences between the UN, ML and AK maps are approximately halved when compared with the differences at the beginning of structure determination, as presented in the previous section (Figs. 1 ▶ and 2 ▶). This is in agreement with the general expectation that with the pro­gress of structure determination the differences between the UN maps and those based on the error estimate function will decrease.

**Figure 4:**
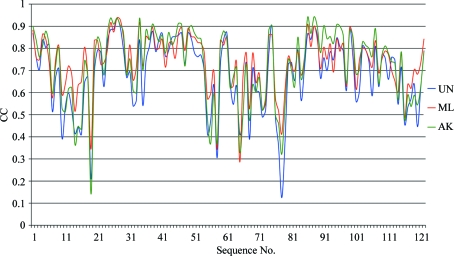
Local map comparison along the chain. UN, ML and AK maps (shown as blue, red and green lines) are compared with the final *F*_model_ map. CCs were calculated for regions belonging to each individual residue of the final structure and are plotted along the whole chain of ammodytin L (PDB code 3dih).

**Figure 5:**
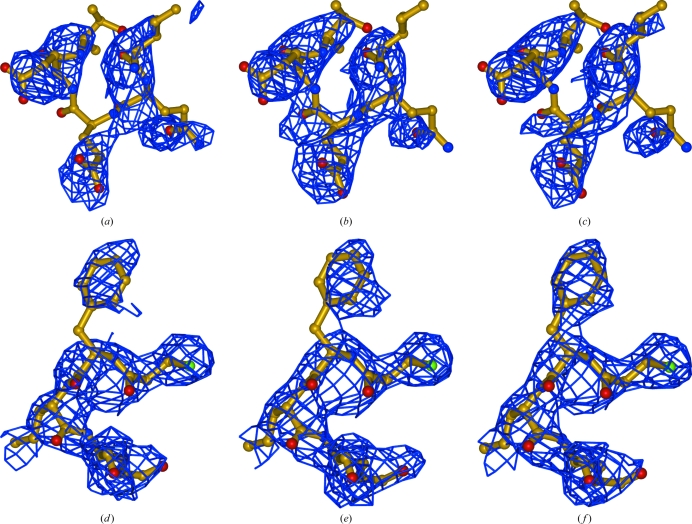
Local map comparison of two regions. (*a*), (*b*) and (*c*) show maps around residues Ile9–Glu13, while (*d*), (*e*) and (*f*) show maps around residues Cys94–Arg98 calculated from a model of ammodytin L (*R* value 0.37, the same as used to prepare Fig. 4 ▶). The final model is shown in stick representation. The maps in (*a*) and (*d*) represent UN maps, those in (*b*) and (*e*) represent ML maps and those in (*c*) and (*f*) represent AK maps. The maps were generated using data at 3.0 Å resolution and are all shown at a 1.0σ contour level.

*Maps from the intermediate phase: local comparison along the chain.* The comparison of maps locally at individual residues along the polypeptide chain revealed that ML and AK maps interchange in their ranking of similarity to the final *F*_model_ map. Since these maps were generated from the same structures from the intermediate phase of structure determination, the fit of the residues along the chain fluctuates and so do the CCs, which differ from map to map. For illustration, the ammodytin L case was chosen. Fig. 4 ▶ shows the residue-based CC of the maps plotted along the whole chain. Visual inspection has confirmed that correlations below 0.5, such as in the regions around residues 19 and 78, do indeed indicate poor similarity of local maps of any kind to the final *F*_model_ map. To illustrate the differences between the AK and ML maps, we have chosen two regions in which the plots of the ML and AK maps are higher than 0.5, not overlapping and ranked differently. In the first region, Ile9–Thr13 (Figs. 5 ▶ *a*, 5 ▶ *b* and 5 ▶ *c*), the ML map provides a more clear representation of the model, whereas in the second region, Ile94–Glu98 (Figs. 5 ▶ *d*, 5 ▶ *e* and 5 ▶ *f*), the AK map is closer to the final map. In the first region the ML map closes the density gap (Fig. 5 ▶ *b*), whereas in the second region the AK corresponds best to the final position of the Phe95, resolving the side-chain ambiguity (Fig. 5 ▶ *f*). These comparisons illustrate that the use of AK maps in combination with ML maps can be useful during model-building procedures and are consistent with past experience.

#### 3.2.3. Maps from the final phase

In the final phase, remaining weak density and dubious map features require interpretation. They are commonly occupied by ligands attached to the macromolecular structure or flexible likely surface-located regions and residue side chains, and exhibit a larger degree of disorder when compared with the core of the structure. We have addressed this issue by re-examining the cathepsin H mini-chain case that initiated kick-map development. The molecular model for this case was generated by using the SA approach with the exclusion of solvent, carbohydrate and mini-chain atoms and yielded an *R* factor of 0.30 at 2.1 Å resolution. We have assumed that this model has lost any memory of the excluded parts and thus represents the structure in the state prior to building the mini-chain residues. The maps resulting from this model are OMIT maps with erased memory and are termed erased-memory maps. To find out which map calculation is most suitable for revealing the correct solution and simultaneously exposing the model contributions and its bias, we attached the eight mini-chain residues to the SA model. The mini-chain residues were built into the same density region in the correct and reverse directions. The two models were refined using an initial 0.3 Å random shift (kick) of each atom coordinate followed by two cycles of positional and *B*-value refinement until convergence. Using these three models (SA, memory erased, correctly and reversely built mini-chain), we have calculated UN, ML and AK maps with the mini-chain residues erased, included and omitted from the map calculations. The direct effects were monitored by comparisons of the maps in the local region in the vicinity of the mini-chain with the final *F*_model_ map (Table 4 ▶).

The erased-memory map of the mini-chain region from the UN map had the lowest CC (0.65), followed by the CCs of the ML (0.68), AK (0.69) and SA (0.69) maps. The same map regions of the correctly built mini-chain have CCs for included as well as omitted maps that were equal or higher than the regions from the erased model, whereas the reversely built mini-chain results in lower or equivalent CCs. The CCs of maps with the correctly built mini-chain are higher than those of the reversely built mini-chain for all the map calculation method used; the best signal is from the AK map. The signal of the OMIT maps is less pronounced; nevertheless, the maps from the reversely built mini-chain are still ranked lower. For comparison, the *F*_model_ maps of the mini-chain region of the correct and reverse models have a CC of 0.66, indicating that there is substantial similarity between them.

None of the OMIT map calculations was capable of eliminating the contribution of the correctly and reversely built mini-chain atoms refined into the structure. However, the comparisons showed that in this case the AK maps provided the strongest signal for the correctly built model and can therefore be a useful alternative to the ML maps. Additionally, this exercise indicates that comparison of the maps calculated from the same model alone may not provide sufficient support for selecting the correct model when multiple interpretations seem possible. In such cases, alternative models should be built, refined and ranked before the correct one can be chosen.

### 3.3. Application to a problematic case

Previous work on the removal of model bias (Terwilliger *et al.*, 2008 ▶) used PDB entry 1zen (Cooper *et al.*, 1996 ▶) determined at 2.5 Å resolution as an example. Residues 6–16 of this structure were in disagreement with the very closely related structure 1b57 (Hall *et al.*, 1999 ▶) determined at 2.0 Å resolution with two copies of the same molecule in the asymmetric unit. The *F*_obs_ − *F*_calc_ map exposed a series of other mis­matches along the chain (Terwilliger *et al.*, 2008 ▶) using the *EDS* server (Kleywegt *et al.*, 2004 ▶). It was shown (Terwilliger *et al.*, 2008 ▶) that the impact of model bias on the map can be removed using an iterative-build OMIT-map approach, whereas other approaches such as ML and SA ML maps of the 2*F*_obs_ − *F*_calc_ type alone failed to resolve the side-chain ambiguity. Visual inspection of both entries together with their maps and their superimpositions revealed that the structures are misaligned in several regions (1–15, 110–114, 194–198, 225–235, 265–271 and 352–356), comprising a total of 46 residues out of 338 (346 in 1b57). Furthermore, the maps also indicate that a solvent molecule with a *B* factor of 2 Å^2^ should have been identified as a metal ion, which was likely to be Zn coordinated by two histidines and two solvent molecules. Since the region 288–340 did not superimpose well owing to real differences between the crystal structures, it was excluded from model superimposition. The remaining 238 matching C^α^-atoms pairs yielded an r.m.s.d. of 0.53 Å, whereas also taking into account the nonmatching C^α^-atom pairs (284) yielded an r.m.s.d. of 2.0 Å. Visual inspection of the maps revealed that Asp15 was built into density belonging to the main chain of the helix N-terminus, thereby causing the one-residue shift. In addition to the density, the chemical environment of the side-chain residues in the region also points to a likely mistake in sequence register. For example, the Thr12 and Asp5 residues were positioned in a hydrophobic environment instead of Ile13 and Ile3. The same kind of error, which cuts the helix one residue too short at its N-terminus, was repeated at position 113 and caused the residue shift in the 110–114 region. To summarize, the 1zen model is partially incorrect (over 10% of residues appear to be displaced from the positions observed in the 1b57 structure).

**Figure 6:**
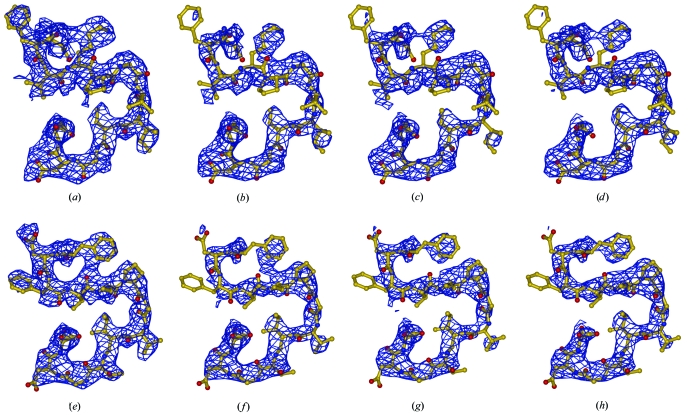
AK maps in structure validation. First- and second-generation AK OMIT maps of the region 1–15 are shown on the background of the 4–16 sequence of the 1zen (*a*–*d*) and 1b57 (*e*–*h*) PDB depositions are shown. The first generation of ML AK and UN AK maps are shown in (*a*) and (*e*) and in (*b*) and (*f*), respectively, and the second-generation ML AL and UN AK maps are shown in (*c*) and (*g*) and in (*d*) and (*h*), respectively. Kick maps were calculated with a single kick size of 0.8 Å and were averaged 100 times. Maps are contoured at 1.2σ.

The question here was, can the AK map approach correctly assign the density cloud of the Phe4 side-chain moiety using the model as deposited without any rebuilding? The procedure, if it is to be successful, must deal not only with the direct and indirect bias of the loop itself but also with the indirect bias of the misplaced atoms spread out through the remainder of the structure. For this purpose, we generated a variety of regional  first- and second-generation OMIT and non-OMIT maps of the UN, ML, UN AK and ML AK 2*F*_obs_ − *F*_calc_ types. Since no correct model of the 1zen deposition is available, we could not compare the maps with the final *F*_model_ map. Instead, we show the maps around the omitted region of interest (residues 3–15) on the background of the 1zen and 1b57 models (Fig. 6 ▶). It turned out that of these maps, only the second-generation UN AK and ML AK maps with kicks between 0.7 and 1.0 Å and with the region 1–15 omitted could resolve the map ambiguity, thus assigning the density cloud to the correct position of the side chain of Phe4 (Figs. 6 ▶ *g* and 6 ▶ *h*) and not to the side chain of Lys8 as present in the 1zen model (Figs. 6 ▶ *a* and 6 ▶ *b*). In addition, the map resulting from the averaging of all ten second-generation AK maps provided the correct answer although with a less clear map. (The CC between the second-generation UN AK and ML AK maps was 0.97 and that between the average of the AK maps with a kick size between 0.1 and 1.0 Å and UN AK map using a 0.8 Å kick was 0.94.)

This case thus demonstrates that AK maps have the potential to remove substantial model bias and can also be used as a valuable structure-validation tool.

## 4. Discussion

**Figure 7:**
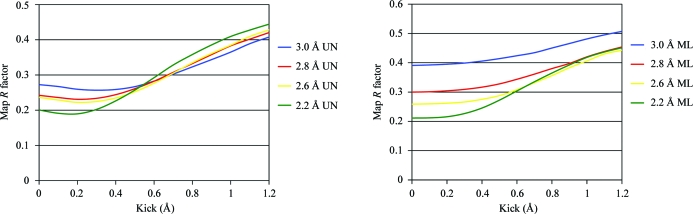
*R* factors of AK maps plotted against kick size. The plots show the *R* factors of the maps of cathepsin H generated for Fig. 2 ▶.

If atoms were kicked and their ensembles were then averaged, it would be expected that the averaged structure would essentially look the same as the original structure. Indeed, when the *F*_model_ structure factors used in map calculation are the sum of the contributions of a series of randomly shifted structures, the resulting AK map reveals no significant improvement when compared with the map calculated from the starting model. This indicates that the map improvement as seen in the AK maps does not solely arise from the averaging of structures, but also contains other error-correction mechanism(s). As indicated above, the averaging of structure factors prior to their scaling to *F*_obs_ eliminates the map improvement. This shows that the change in phases must be coupled with individual scaling of *F*_model_ to *F*_obs_ in order to achieve the desired effect. The change of the phases is similar to the SA concept, which calculates maps from an ensemble of structures, while application of modified scaling coefficients exhibits similarity to the ML weighting scheme (Read, 1986 ▶; Pannu & Read, 1996 ▶). The lower noise of the AK maps also makes them more similar to the ML maps. Comparison of optimal kick size and ML estimates of coordinate error (Table 2 ▶) further confirms the analogy between the two approaches. The ML error estimate of the coordinates does not necessary coincide with the best model bias-removal value, although the values in Table 3 ▶ indicate that they become rather close. Although averaging of ten AK maps generated with different kick sizes in principle successfully eliminates the need for the kick-size estimate, in extreme cases when the map is seriously biased by the model the application of a larger kick size such as 0.8–0.9 Å may be crucial. However, there are two differences between the AK and SA approaches. Firstly, during the SA procedure parts of the structure may drift away by several angstroms (multi-start SA has the potential to model multiple alternative conformations), whereas kicking keeps atoms within the specified frame. Secondly, the violations of chemical terms are severe in kicked structures (the r.m.s. deviation of bond lengths from their targets is usually only slightly lower than the kick specified), whereas SA molecular models remain chemically reasonable during and after the procedure, which preserves a higher degree of direct and indirect atomic interactions and consequent coupling of model errors. Because of the shifting of atoms around the starting point with a predefined maximal shift size, the concept of kicking is more similar to the ML approach since they both address the random model errors, whereas SA has the potential to shift parts of the model over larger distances and also has the potential to fix systematic errors. Interestingly, however, the phase errors of AK maps are slightly higher than those of the input model, indicating that the lower noise of the maps cannot be directly accounted for by the phase improvement (data not shown). However, looking at the *R* values obtained by Fourier transformation of each AK map (Fig. 7 ▶) it appears that all *R* values of UN AK as well as ML AK maps start lower than the *R* value of the initial models (Table 3 ▶) and then increase in a kick-size-dependent manner. (Shallow minima are observed in the UN AK map series.) The average *R* factor of a series of AK maps is much lower than the sum of the series (individual kick maps have significantly higher *R* factors), indicating the importance of the weighting-scheme contribution to the success of the AK approach.

As shown above with the 1zen case, second-generation AK OMIT maps were able to clarify a problematic region in the density without the need for model rebuilding. Omitting the problematic region of the structure appeared to be essential for reducing the direct model bias, while kicking in combination with omission of the parts inconsistent with the first-generation AK maps resulted in sufficiently reduced indirect model bias. With this, the AK maps approach exhibited a potential similar to the achievements of the iterative-build OMIT-maps approach (Terwilliger *et al.*, 2008 ▶). The latter maps are, in comparison with the AK map approach, rather complex and computer-time-demanding procedures. Hence, the AK map approach is yet another contribution to the series of map calculations dealing with model-bias removal such as OMIT, SA OMIT maps, composite OMIT maps (Bhat & Cohen, 1984 ▶; Bhat, 1988 ▶; Hodel *et al.*, 1992 ▶; Brünger *et al.*, 1998 ▶) and the prime-and-switch phasing approach (Terwilliger, 2004 ▶). As such, AK maps can also be used in *de novo* crystal structure determination. The potential revealed here suggests that AK maps are a fast and simple approach that may offer considerable help during macromolecular crystal structure determination.

## Figures and Tables

**Table 1:** Data and model statistics used in tests

PDB code, molecule	Resolution (Å)	No. of atoms	Data completeness (%)	No. of reflections	*R* factors (work/free)
2oct, stefin B tetramer	1.4	1703	100	37125	0.18/0.23
8pch, cathepsin H	2.1	1982	98	15946	0.20/0.25
3dih, ammodytin L	2.6	1007	96	5323	0.16/0.23
2ahn, cherry allergen	1.3	1887	86	49825	0.12/0.15
2fy2, choline acetyltransferase	2.25	5128	98	32854	0.21/0.23
1twl, inorganic pyrophosphatase	2.2	1410	100	9314	0.20/0.26

**Table 2:** Models at various stages of refinement Intermediate models saved during the course of cathepsin H structure determination were refined against the data in various resolution ranges. The table lists models with corresponding *R* values (model, average of series of UN AK and ML AK maps), the number of equipositioned C^α^ atoms with the final model, r.m.s. deviations from the final structure, optimal kick size and ML estimates of coordinate errors. R.m.s. values were calculated between pairs of equipositioned C^α^ atoms of the final and intermediate models. The number in parentheses shows the number of nonmatching C^α^ atoms with a distance larger than the cutoff of 2.0 Å.

Intermediate models	Resolution (Å)	*R* factor model	*R* factor UN AK	*R* factor ML AK	C^α^ pairs	C^α^ r.m.s. (Å)	Optimal kick (Å)	ML estimated coordinate error (Å)
Model 1	3.0	0.43	0.26	0.42	220 (29)	0.91	1.0	1.14
Model 2	2.8	0.37	0.26	0.33	220 (23)	0.65	0.8	0.72
Model 3	2.6	0.31	0.25	0.29	228 (12)	0.45	0.6	0.58
Model 4	2.2	0.25	0.25	0.27	228 (1)	0.27	0.4	0.37

**Table 3:** Maps in comparison with the final *F*_model_ map Map CCs for the specified map were calculated between final *F*_model_ and UN, ML, AK and SA maps. One of the SA models at a final resolution of 2.6 Å (ammodytin L) and 2.1 Å (cathepsin H) was used.

Map	Ammodytin L	Cathepsin H
UN	0.81	0.78
ML	0.84	0.80
AK	0.84	0.81
SA	0.85	0.81

**Table 4:** The effect of the model contribution and its bias CCs were calculated between *F*_model_ of the final map and UN, ML and averaged AK maps of an OMIT and non-OMIT working model of the mini-chain region. The maps were calculated for the model with the mini-chain erased, correctly and reversely built and refined.

	Mini-chain region				
		Correct		Reverse	
	Erased	Included	OMIT	Included	OMIT
UN	0.65	0.69	0.65	0.63	0.62
ML	0.68	0.72	0.69	0.68	0.67
AK	0.69	0.75	0.7	0.68	0.67
SA	0.69	—	—	—	—
